# Changes in Frequency-Doubling Perimetry in Patients with Type I Diabetes prior to Retinopathy

**DOI:** 10.1155/2013/341269

**Published:** 2013-11-11

**Authors:** Isabel Pinilla, Antonio Ferreras, Miriam Idoipe, Ana I. Sanchez-Cano, Diana Perez-Garcia, Laura X. Herrera, Maria J. Pinilla, Emilio Abecia

**Affiliations:** ^1^Department of Ophthalmology, Hospital Clinico Universitario Lozano Blesa, Aragon Health Sciences Institute, 50009 Zaragoza, Spain; ^2^Miguel Servet University Hospital, Aragon Health Sciences Institute, 50009 Zaragoza, Spain; ^3^Department of Applied Physics, Zaragoza University, 50009 Zaragoza, Spain; ^4^Department of Cardiology, Hospital Clinico Universitario Lozano Blesa, Aragon Health Sciences Institute, 50009 Zaragoza, Spain

## Abstract

*Purpose*. To evaluate the ability of frequency-doubling technology (FDT) perimetry in detecting visual field defects in young adults with type I diabetes prior to retinopathy or with minor retinovascular changes. *Methods*. This comparative cross-sectional study included 30 healthy subjects and 73 age-matched patients with type I diabetes mellitus. All subjects underwent a full ocular examination including an FDT with the threshold C-20-5 strategy. Only one eye per subject was randomly included in the statistical analysis. FDT results and time to perform the test were compared between the groups. *Results*. The mean age was 27.1 years in the control group and 26.6 years in the diabetic group (*P* = 0.875). The mean period from the onset of diabetes was 12.6 ± 6.7 years, while minimal retinovascular changes were observed in 18 eyes. Mean deviation of FDT did not differ between the groups. Although global indices of FDT were within normal limits, pattern standard deviation of FDT was higher in the diabetic group (*P* = 0.035). The area under the receiver operating characteristic curve was 0.647 for pattern standard deviation of FDT (standard error = 0.052; *P* = 0.017). *Conclusion*. FDT can detect retinal dysfunctions in diabetic patients prior to the onset of significant vascular complications.

## 1. Introduction

Diabetic retinopathy is the major cause of visual loss in the diabetic population, although it is not the only aspect of visual dysfunction in these patients. There is accumulating evidence indicating that the impairment of retinal function precedes the earliest signs of vascular complications. Also, functional defects have been reported in animal models of diabetic retinopathy prior to the occurrence of any clinically visible changes [1, 2]. Such defects are attributed to a decrease in nerve function and consist of alterations in oscillatory potentials, pattern electroretinogram (ERG), multifocal ERG, S-cone ERG, and paired-flash ERG [3–10]. Other changes include reductions in dark adaptation, visually evoked responses, contrast sensitivity, and optic nerve conduction velocity [11–13]. Additionally, abnormal colour sensation [14], prolongations in visually evoked response latencies [15–22], and defects in the retinal nerve fiber layer [23] have been detected at stages preceding detectable retinopathy. 

Some years ago, frequency-doubling technology (FDT) perimetry was introduced, a method for assessing contrast threshold based on the spatial frequency-doubling illusion that occurs when a low spatial frequency sine wave grating is counterphased at a high temporal rate, resulting in the grating's appearing to have twice its original spatial frequency [24–28]. Frequency-doubling illusion does not depend on contrast reversal and any rapid temporal variation will do either translation or pattern onset. Thus, localized visual field defects are tested as malfunctions of the temporal contrast behaviour and may contribute to the results obtained with the FDT perimeter. 

White et al. [29] suggested that a cortical loss of temporal phase discrimination is the principal cause of the illusion and proposed that the mechanisms underlying the illusion resemble those underlying the detection of full-field flicker, which appears to be accomplished through the magnocellular pathway. Therefore, FDT can be abnormal not only in patients with visual field defects, such as those found in glaucoma, but also in patients with impaired contrast sensitivity. The threshold contrast, at which a pattern of stripes is just visible, is a measure of a different visual function that can pick up deficits in diverse pathologies including retinal diseases [11, 30–32]. The reduction in contrast sensitivity found in diabetic patients can be a source of abnormal FDT [33].

To our knowledge this is the first study aimed to evaluate the efficacy of FDT in detecting functional impairment of vision in patients with type I diabetes without retinopathy or with minor retinovascular changes.

## 2. Materials and Methods

The comparative cross-sectional study protocol was approved by the ethics committee of the Miguel Servet University Hospital and written consent was obtained from all participants. 

Thirty consecutive eyes of 30 healthy young adults and 73 consecutive eyes of 73 patients with type I diabetes mellitus that met the inclusion criteria were prospectively enrolled in the study. Control eyes were selected from patients referred for refraction and routine exploration without abnormal ocular findings, hospital staff, and relatives of patients of our hospital. Diabetic patients were recruited from those referred for annual diabetic retinopathy screening. 

The following criteria were met in both groups: age between 15 and 45 years, no cardiac diseases or uncontrolled arterial hypertension (over 130/80 mmHg), best-corrected visual acuity of 20/30 or better (Snellen), Goldmann applanation tonometry lower than 20 mmHg, refractive errors less than 5 spherical diopters and 2 cylinder diopters, and transparent ocular media (nuclear color/opalescence, cortical or posterior subcapsular lens opacity <1) according to the lens opacities classification system (LOCS) III [34]. Subjects with previous intraocular surgery, history of ocular or neurologic disease, or current use of a medication that could affect visual field sensitivity were excluded. One eye per patient was randomly selected for the study.

Control eyes presented normal ocular findings. The funduscopic examination of diabetic patient required, as a selection criterion for this study, the absence of retinopathy or minimal changes. The presence of five or more microaneurysms in each eye was an exclusion criteria; no patient had received previous laser treatment. The level of retinopathy was estimated according to the early treatment diabetic retinopathy study (ETDRS) guidelines [35]. 

All of them had a full ophthalmologic examination: clinical history, visual acuity, biomicroscopy of the anterior segment using a slit lamp, intraocular pressure, indirect ophthalmoscopy (evaluation of peripheral retina), and stereoscopic ophthalmoscopy of the posterior segment. Additionally, diabetic patients underwent a blood extraction within 3 months from the subject's date of enrolment into the study to measure the HbA1c level.

At least 2 digital color fundus photographs with a 45-degree field of view were acquired through dilated pupils (1% tropicamide eye drops; Alcon Laboratories Inc., Fort Worth, TX). One of the fundus images was centered on the fovea, while the other was centered on the optic nerve head. The images were evaluated by two independent observers (Isabel Pinilla and Emilio Abecia) who were masked about the patients' identity and the status of disease. Any disagreement was resolved by consensus.

FDT was performed under low ambient light using the original frequency-doubling perimeter (Carl Zeiss Meditec, Dublin, CA). The full-threshold C-20 strategy was used and perimetric data were analyzed with Windows ViewFinder 1.0 software. Trial lenses and patches were not applied, but subjects wore their own corrective lenses. Conditions for reliable test were no more than 1/6 fixation losses (16%), no more than 1/6 false positive (16%), and no more than 1/3 false negative responses (33%); otherwise, visual field tests were repeated. The criterion of abnormal points for FDT was defined by the presence of at least 5 points lower than *P* < 5%, 2 points lower than *P* < 2%, or 1 point lower than *P* < 1% on pattern deviation plot. The abnormal locations could be anywhere within the FDT field [36]. Mean deviation (MD) and pattern standard deviation (PSD) results and time of performance of the test were also included in the statistical analysis. Every subject completed the FDTs prior to any other exploration, and if the perimetry had to be repeated, it was completed in a different day to avoid a fatigue effect. 

All statistical analyses were performed using the IBM SPSS (version 21.0; IBM Corporation, Somers, NY) and MedCalc (version 12.7; MedCalc software, Mariakerke, Belgium) statistical software. The Kolmogorov Smirnov test was used to check for a normal distribution of the data. Differences between both groups were tested using Student's *t*-test when data followed normal distributions. The probability level at which the null hypothesis was rejected was set at *P* < 0.05. The receiver operating characteristic (ROC) curves were plotted for the MD and PSD of FDT (bootstrap replications: 1000). The cut-off points were calculated by the MedCalc software as the points with the best sensitivity-specificity balance. 

## 3. Results

### 3.1. Results Are Expressed as Mean ± Standard Deviation

#### 3.1.1. Patient Data

**Figure 1:**
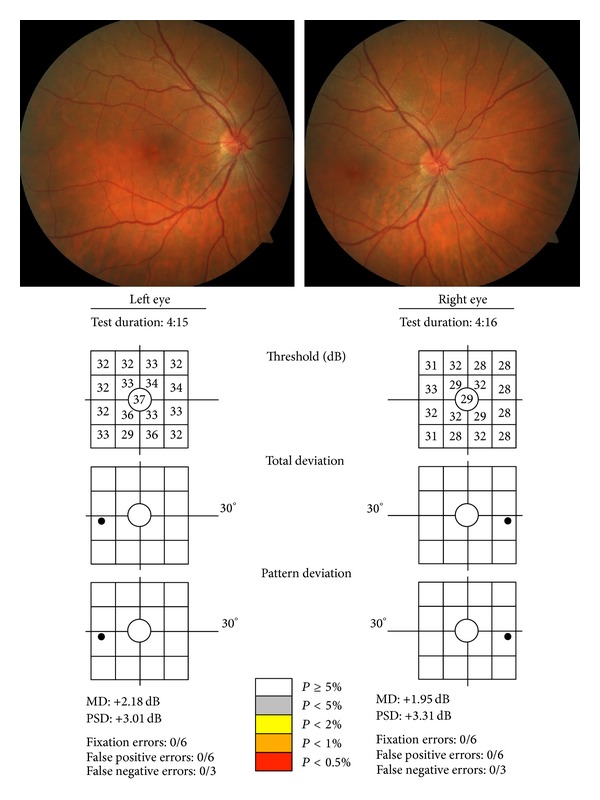
Fundus photographs (one centered on the fovea and the other in the optic disc) and FDT outcome of the same patient with type I diabetes and minor retinovascular changes.

The control group comprised 12 women and 18 men. Their mean age was 27.1 ± 9.1 years (range: 15 to 44 years; Table 1). The diabetic group included 30 women and 43 men; their mean age was 26.6 ± 8.3 years (range: 15 to 40 years).  Figure 1 shows the funduscopic appearance of a diabetic patient with no retinal findings and its FDT results. The mean period from the onset of diabetes was 12.62 ± 6.7 years and the mean HbA1c was 7.78 mg/dL (Table 2). In 32 of them (43.8%), the mean HbA1c value was under 7.5 mg/dL and 56.1% (41 patients) had it over this level. Better HbA1c control was achieved in patients with higher number of insulin injections per day. According to ETDRS severity criteria applied to our fundus photographs [35], 55 patients had no retinopathy (level 10, 75.3%) and 18 patients (24.7%) had microaneurysm formation only (level 20). No additional changes were detected. Clinical data of diabetic patients are presented in Table 2. Patients with less than 15 years of history of diabetes were 43 (58.9%) and 30 had more than 15 years since the diabetic onset. The age of the diabetic patients not affected by ocular changes was lower (*P* < 0.001) than the age of the patients with lesions (25.35 ± 7.4 years versus 32.3 ± 8.3 years, resp.). There were also differences (*P* < 0.001) in the age of onset of the diabetes between patients without funduscopic lesions and those with minor retinovascular changes: the mean diabetes duration was 11.06 ± 5.8 years in subjects without retinal damage and 20.2 ± 4.2 years in patients with minor lesions. 

No significant difference was found for the factors affecting retinal blood flow such as level of blood pressure, cigarette smoking, and rheologic factors, as aspirin use, between the groups. 

#### 3.1.2. Visual Field Data: Altered Points, MD, and PSD

MD of FDT was not different (*P* = 0.13) between the control and diabetic group (0.25 ± 1.5 dB and −0.04 ± 2.1 dB, resp.). However, PSD of FDT differed between the groups (3.43 ± 0.4 versus 3.78 ± 0.9 in diabetic group; *P* = 0.035). 

Two subjects (6.7%) of the control group and 15 (20.5%) of the diabetic patients showed abnormal FDTs based on the criteria of altered points. No significant differences were observed between both groups (*P* = 0.09). In the diabetic group, 12 (21.8%) of the abnormal FDT were from patients with no retinopathy and 3 (16.7%) with minimal vascular changes. No significant differences were observed between the two diabetic groups (*P* = 0.82). Abnormal perimetries depending on the criterion of altered points showed a significant higher PSD value (*P* = 0.004) than normal tests (4.37 ± 0.5 and 3.62 ± 1.0, resp.).

We examined whether the age of diabetes onset was related to the FDT findings. Patients with less than 15 years of the disease had a PSD of 3.72 ± 0.63, while those with more than 15 years of the disease had a PSD of 3.84 ± 1.16 (*P* = 0.56). There was no relationship between the HbA1c level and the mean PSD values (3.69 ± 0.7 and 3.85 ± 1.1 in patients with HbA1c levels lower and higher than 7.5, resp.). Neither the number of insulin injections per day was related to the PSD value. 

**Figure 2:**
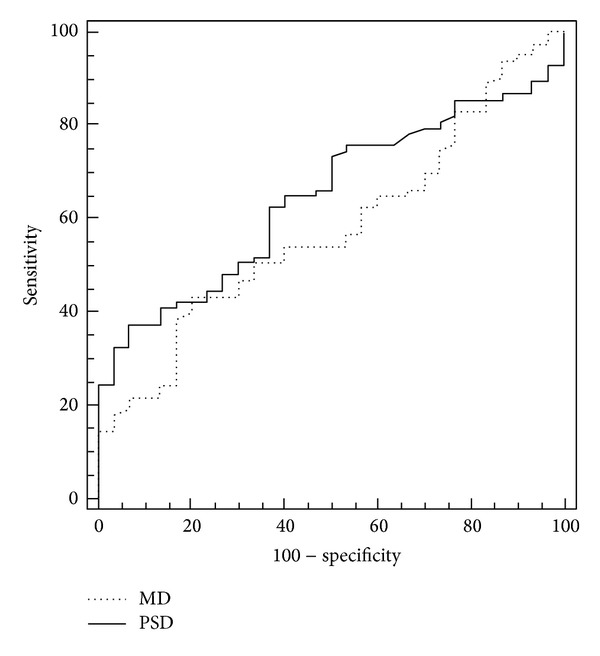
Receiver operating characteristic (ROC) curves for MD and PSD of FDT between healthy and diabetic patients.

The MD of FDT had an AUC of 0.580 (95% confident interval (CI): 0.467–0.692; standard error = 0.057; *P* = 0.198) while the PSD of FDT had an AUC of 0.647 (95% CI: 0.545–0.750; standard error = 0.052; *P* = 0.017; Figure 2). The best sensitivity-specificity balance for PSD of FDT was 37.3%–93.3%, respectively (cut-off point > 3.99).

#### 3.1.3. Test Time

There were no differences in the test times between both groups. The control group needed a mean time of 4.33 ± 0.3 minutes to perform the perimetry and the diabetic group required 4.37 ± 0.4 minutes (*P* = 0.69). Nevertheless, the time to perform the FDT tests depended on the years which passed since the diagnosis of the disease. Patients with less than 10 years following diagnosis of diabetes required a time of 4.21 ± 0.4 minutes, while those with more than 10 years required a time of 4.42 ± 0.5 minutes (*P* = 0.045). 

## 4. Discussion

Visual dysfunctions occur in diabetic patients before obvious vasculopathy. Consequently, the vascular aberrations per se are not necessarily the cause of the visual losses. Since retinal neurons transduce visual signals, it follows that the loss of neuronal function within the retina circuitry is likely to lead to visual impairment. Diabetes is known to have a direct effect on retinal neurons and, as a result, alterations in neuronal function could occur soon after the beginning of the diabetes [1, 2]. These neuropathological changes themselves may contribute to the development of the diabetic retinopathy. Abnormalities in a wide range of electrophysiological tests have been found before vascular anomalies were detected. The present study explored whether the FDT would be useful for the individual patient in detecting such changes in neuronal function. 

FDT is a quick and efficient way of screening visual field with a very good sensitivity and specificity [28]. This perimetry is most likely a probe of contrast sensitivity of the magnocellular pathway [29]. Contrast sensitivity is known to be affected in diabetic patients and, therefore, FDT can be a promising psychophysical test candidate for the detection of not only glaucoma [37] but also other diseases such as diabetic retinopathy. 

The C-20-5 strategy tests the central 20° (10° × 10° targets), 4 per quadrant within the central 20°, with one smaller central target (5° diameter circle) projected on the macular region. In our study, we used 45° fundus photographs centered on the fovea and the optic disc. Consequently, they should cover the area of the retina assessed by FDT. We did not find changes in the number of altered FDT tests. This suggests that the diabetic patient might not already have detectable visual field defects. Thus, it is important to consider that the abnormality criterion for FDT was based on glaucoma patients [36]. Hence, there will be a need for a new definition for patients with diabetes, who are likely to have different visual field defects than patients with glaucoma. 

Although MD was similar between diabetic and normal groups, there was a statistically significant difference in PSD values. This suggests that localized diminutions of retinal sensitivity occur in diabetic patients, prior to the appearance of retinal lesions. It is relevant to note that the median age of the diabetic group was 26 years; at this age no other optic nerve pathologies are expected to be found. No differences were observed between patients with normal or minimal fundus changes. These can be explained for two reasons: (1) the low number of patients affected with background retinopathy and (2) the minimal changes they were showing. To know if these changes can predict the appearance of retinal vascular abnormalities in the future, a prolonged follow-up should be performed. It is likely that those patients whose contrast sensitivity was impaired later would show background retinopathy. Alternatively, the test results may suggest the possibility of an ischemic involvement; this could be controlled by taking into account angiographic findings. However, screening normal patients with angiographies should be avoided if possible. Parikh et al. [38] reported the possibility of using the FDT as a way of detecting sight-threatening diabetic retinopathy. One key difference from the present study is that Parikh et al. included patients with advanced retinopathy states in their study. Although their findings remain in compliance with the ability of FDT in detecting ischemic states, a crucial use of the FDT should be the ability of detecting potential funduscopic lesions prior to their occurrence, therefore allowing preventive measures to be taken. 

Realini et al., using the C-20-5 screening algorithm of the FDT perimeter, have found that diabetes can affect glaucoma screening using FDT testing and recommended the exclusion of patients with cataract and/or diabetes from this screening, suggesting that the specificity of the screening would improve [33]. They used a suprathreshold strategy derived from the fifth percentile of normal values, but nowadays this algorithm is not available in the software for the commercial version of this device. Our study has shown results based on a threshold strategy that can be performed in the original commercialized FDT perimeter. 

Other authors reported that Matrix and scanning laser polarimetry with variable corneal compensation (GDx VCC) could be useful to identify early retinal impairment in patients with type I diabetes mellitus and without signs of retinal vasculopathy [39]. The Humphrey Matrix perimeter (Carl Zeiss Meditec) allows FDT algorithms similar to those of conventional perimetry. In spite of this innovation, the original FDT perimeter cannot be considered outdated because each of these instruments has a different purpose. 

Bell et al. [40] used the multifocal pupillographic perimetry to evaluate changes in patients with early type 2 diabetes. They used transient stimuli containing low spatial frequencies (less than 2 cycles/degree) and found that some retinal damage was present in the near absence of visible diabetic retinopathy, especially in those patients with more than 10 years of diabetes.

FDT is a fast examination, ideal as a screening approach, with an analysis time shorter than other strategies used in standard automated perimetry. FDT testing is commonly achieved in less than 1 minute for the suprathreshold algorithm and less than 5 minutes for the threshold detecting mode. This fact makes FDT a good option for testing visual field in all kind of subjects, even children. Patel et al. [41] found that subjects with normal visual fields completed the screening C-20-1 FDT examination within 90 seconds, and times of performance longer than 90 seconds can be considered as abnormal FDT. In our study, no significant differences were observed for the test time between normal subjects and the diabetic group. However, for the C-20 threshold algorithm the time of the perimetry cannot be applied as a marker of disease, because obviously the way of testing the visual field points is different for a threshold than for a suprathreshold mode. 

A major problem of some psychophysical tests used for glaucoma screening such as FDT or short-wavelength automated perimetry is the high false positive rate that would reduce the specificity [42]. It is important to take into consideration that patients with ocular pathology like cataracts and systemic diseases such as diabetes, with or without retinal manifestations, can interfere in some glaucoma screening tests including FDT. Cataract is known to alter the contrast sensitivity perception, and it has been demonstrated that patients who underwent cataract extraction indeed improved their retinal sensitivity [43]. 

## 5. Conclusions

Diabetic patients without retinopathy or with minor retinovascular changes have alterations in FDT threshold test with a diminution of the sensitivity. The findings suggest the occurrence of retinal dysfunctions prior to the onset of diabetic retinopathy. The test could be considered as a fast way to determine whether there is a diminution in retinal sensitivity in these patients.

## Figures and Tables

**Table 1:** Demographic data and results of FDT in both study groups. *P* < 0.05 was considered statistically significant (in bold print).

	Control		Diabetics		*P* value*
	Mean	SD	Mean	SD	
Age (yrs)	27.1	9.14	26.6	8.3	0.875
BCVA (Snellen)	0.93	0.1	0.94	0.1	0.645
IOP (mmHg)	14.93	2.2	14.72	2.4	0.680
FDT MD (dB)	0.25	1.52	−0.04	2.05	0.130
FDT PSD	3.43	0.39	3.78	0.95	**0.035**
Test time (min)	4.33	0.27	4.37	0.44	0.692
*n*	30		73		

*Student's *t*-test.

BCVA: best-corrected visual acuity; IOP: intraocular pressure; FDT: frequency-doubling perimetry; MD: mean deviation; PSD: pattern standard deviation; SD: standard deviation.

**Table 2:** Clinical data of diabetic patients.

	Mean	SD	Minimal value	Maximal value
HbA1c last 3 months	7.78	1.33	5.5	11.6
HbA1c last year	7.76	1.35	4.5	10.8
Number of insulin injections per day	3.10	0.63	1	5
Number of insulins	2	0.55	1	4

SD: standard deviation.
